# DNE1 scissorhands: How the power of *omics* sheds light on the control of mRNA decay

**DOI:** 10.1093/plcell/koae219

**Published:** 2024-07-24

**Authors:** Margot Raffeiner

**Affiliations:** Assistant Features Editor, The Plant Cell, American Society of Plant Biologists; Faculty of Biology and Biotechnology, Ruhr University Bochum, Bochum 44801, Germany

Eukaryotes are true organizational talents when it comes to maintaining cellular functions. Here, regulation is key. Every conceivable process, including gene transcription, mRNA processing, and translation, but also mRNA and protein decay, is strictly regulated. This tight control allows the establishment of a pool of functional mRNAs and proteins that are constantly available to fine-tune cellular responses.

mRNA homeostasis reflects a balance between mRNA synthesis and decay, where rapid degradation of certain mRNAs effectively preserves RNA metabolism, gene expression, and the fidelity of translation (Li et al. 2018). Plants employ 2 conserved mRNA degradation mechanisms, the 5′-3′ RNA degradation catalyzed by the main 5′-3′ exoribonuclease XRN4, and the 3′-5′ RNA degradation mediated by 3′-5′ exoribonucleases including SOV, the RNA exosome, and their auxiliary factors. To initiate 5′-3′ mRNA degradation, a limiting step is the removal of the methylated guanosine cap at the 5′ end of mRNAs. Decapping is performed by the decapping enzyme DECAPPING 2 (DCP2) together with the decapping enhancers DCP1 and VARICOSE (VCS). In addition to the removal of defective or superfluous mRNAs, the aforementioned mRNA decay factors also protect endogenous mRNAs from silencing, preventing developmental defects and even lethality. Here, decapping again marks RNA substrates to be rapidly degraded, thereby avoiding the accumulation of mRNA-derived small interfering RNAs (siRNAs) that would otherwise be recognized by the plant RNA silencing machinery (Gregory et al. 2008; Li et al. 2018).

In Arabidopsis (*A. thaliana*), the endoribonuclease DCP1-ASSOCIATED NYN ENDORIBONUCLEASE 1 (DNE1) interacts with DCP1 and VCS to stimulate RNA degradation and is required for plant development (Schiaffini et al. 2021). A subset of mRNA targets of DNE1 was previously identified by an RNA degradome analysis suggesting that it likely regulates various additional cellular processes (Nagarajan et al. 2023). However, its full target spectrum and how it interplays with other RNA decay mechanisms remains to be unraveled.

In this issue, **Aude Pouclet and colleagues (Pouclet et al. 2024)** present a multi-transcriptomics approach that allowed the identification of a more complete set of DNE1 targets, as well as shedding light on its coordinated action with the decapping factors to regulate mRNA fate. The authors used 2 complementary high-throughput sequencing (HTS) methodologies, HyperTRIBE and genome-wide mapping of uncapped and cleaved transcripts (GMUCT), enabling them to specifically identify mRNAs that are bound and cleaved by DNE1 (Rahman et al. 2018; Willmann et al. 2014). This also revealed that DNE1 preferentially targets the coding sequence (CDS) rather than the 5′ or 3′ extremities. The degradome analysis by GMUCT was performed in Arabidopsis mutants lacking either the exoribonuclease XRN4 or both XRN4 and DNE1, a key genetic trick to identify mRNAs that are directly cleaved by DNE1, thereby producing DNE1-dependent RNA degradation intermediates (see Figure A). This analysis further allowed the identification of nucleotide cleavage preferences and other mRNA features that make them optimal DNE1 targets. Interestingly, the authors also identified transcripts that produced more decapped intermediates in the absence of DNE1, supporting the idea that DNE1 and the decapping complex have coordinated functions (see Figure B). To investigate this interplay more thoroughly, transcriptomic analyses were performed on different mRNA decay mutants, revealing deregulated candidates that pave the way for a better understanding of the importance of DNE1, DCP2, and XRN4 in this process. Last but not least, Pouclet et al. also identified further mRNA targets of XRN4, DCP2, and DNE1 by looking at mRNA-derived siRNA signatures that accumulate whenever these mRNA decay factors were missing.

**Figure.**
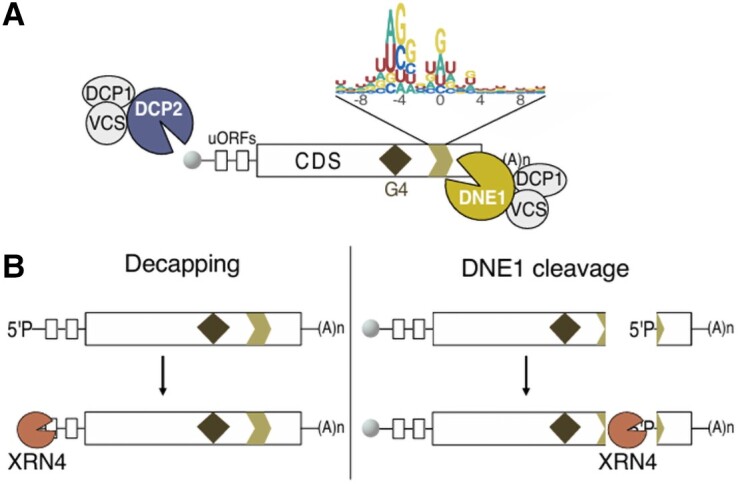
DNE1 and DCP2 coordinately regulate mRNA fate. **A)** Endoribonuclease activity of DNE1. DNE1 preferentially cleaves mRNA targets in their coding sequence (CDS) on sites with specific nucleotide composition. Features enriched in DNE1 targets (RNA G-quadruplex [RNA-G4] and upstream open reading frames [uORFs]) are also depicted. Additionally, it shows that decapping by the DCP2-containing decapping complex takes place at the 5′ UTR of mRNA targets. **B)** Decapping and DNE1 cleavage produce ′ monophosphate mRNA fragments (5′P) labels on their targets and therefore allow the degradation of RNA intermediates by XRN4 starting from different parts on the mRNA. Adapted from Pouclet et al. (2024), Figure 7.

In summary, the authors present an extensive list of mRNA decay factor targets, where some were commonly identified in all HTS methods used, while others needed a specific method to be uncovered. This indicates that with every method, a distinct portion of the transcriptome can be captured and likely points toward the existence of different sets of DNE1 targets and different downstream consequences for mRNA fate.

Overall, this study demonstrates how powerful the combination of different large-scale transcriptomic methodologies is to overcome the limitations of each of the single methods. This approach allows an accurate investigation of important factors involved in the regulation of mRNA fate, plant development, and beyond.
